# Past, present, and future of the Living Planet Index

**DOI:** 10.1038/s44185-023-00017-3

**Published:** 2023-06-01

**Authors:** Sophie E. H. Ledger, Jonathan Loh, Rosamunde Almond, Monika Böhm, Christopher F. Clements, Jessica Currie, Stefanie Deinet, Thomas Galewski, Monique Grooten, Martin Jenkins, Valentina Marconi, Brett Painter, Kate Scott-Gatty, Lucy Young, Michael Hoffmann, Robin Freeman, Louise McRae

**Affiliations:** 1 Institute of Zoology, Zoological Society of London (ZSL), London, UK; 2 School of Anthropology and Conservation, University of Kent, Canterbury, UK; 3 WWF Netherlands – World Wide Fund for Nature, Zeist, Netherlands; 4 Global Center for Species Survival, Indianapolis Zoo, Indianapolis, USA; 5 School of Biological Sciences, University of Bristol, Bristol, UK; 6 WWF Canada – World Wildlife Fund Canada, Toronto, Canada; 7 Institut de recherche pour la conservation des zones humides méditerranéennes, Tour du Valat, Arles, France; 8 Independent researcher, Cambridge, UK; 9 Environment and Climate Change Canada (ECCC), Government of Canada, Gatineau, Canada; 10 WWF UK – World Wide Fund for Nature, Woking, UK; 11 Conservation and Policy, Zoological Society of London (ZSL), London, UK

**Keywords:** Conservation biology, Biodiversity

## Abstract

As we enter the next phase of international policy commitments to halt biodiversity loss (e.g., Kunming-Montreal Global Biodiversity Framework), biodiversity indicators will play an important role in forming the robust basis upon which targeted, and time sensitive conservation actions are developed. Population trend indicators are one of the most powerful tools in biodiversity monitoring due to their responsiveness to changes over short timescales and their ability to aggregate species trends from global down to sub-national or even local scale. We consider how the project behind one of the foremost population level indicators - the Living Planet Index - has evolved over the last 25 years, its value to the field of biodiversity monitoring, and how its components have portrayed a compelling account of the changing status of global biodiversity through its application at policy, research and practice levels. We explore ways the project can develop to enhance our understanding of the state of biodiversity and share lessons learned to inform indicator development and mobilise action.

## Introduction

The Living Planet Index (LPI) (Box 1) was first proposed as a means of evaluating environmental change, particularly by tracking trends in global biodiversity, a quarter of a century ago^1^. At that time, although there was mounting evidence of anthropogenic impacts on nature^2^, there were very few indicators of the state of biodiversity or ecosystems at a global, or even regional scale. The initial version of the LPI, based on trends in vertebrate populations and forest cover, indicated that biodiversity was in decline globally^1^. A successful response to what is now widely recognised as a global biodiversity crisis^3–6^ will involve transformative changes in the way humans use the planet’s resources^7–9^, and widespread intergovernmental action^10^ supported by actions from business, civil society groups and local communities^11^. To this end, governments have agreed ambitious targets^8,9^ (such as the Convention on Biological Diversity (CBD) Kunming-Montreal Global Biodiversity Framework (K-M GBF)^12^ and the United Nations Sustainable Development Goals (SDGs)^13^) to put nature on a path to recovery. However, to track progress towards targets down to the national level we need meaningful and reliable biodiversity indicators, generated from high quality and large-scale data^9,14^. As such, the development of biodiversity indicators has become an increasing focus in conservation science^15–17^, particularly to ensure they are fit for purpose as tools for management and policy, as well as to improve the representation of the underlying data beyond well-studied taxa and regions.

Within this review we chart the history, progression, and applications of the LPI project (Box 1). We review the LPI as a tool for public engagement and outreach, policy, and to drive further research, and analyse citation data to explore other applications of the LPI. We discuss challenges faced in maintaining a large biodiversity dataset and in current uses of the LPI. Finally, we look to the future and propose how the LPI project could evolve by enabling global collaboration to strengthen the indicator, harnessing new technologies for collecting population data, and developing new analysis to better understand the relationships between drivers and wildlife population trends.

Box 1. The Living Planet Index project The Living Planet Index project (the index, methodology, and database) and its secondary outputs (methods papers and R code, dataset and website, global index, and subset indices) have had wide-ranging applications within the fields of biodiversity monitoring and research, as well as across policy, education, and outreach.

The Living Planet Index (LPI) is a biodiversity indicator which tracks trends in the relative abundance of wild vertebrate populations (where population is defined as a single species in a defined location rather than the biological definition). Relative abundance captures how populations are changing over time on average in comparison to a reference point, or “baseline” (the LPI uses 1970). It is often described as analogous to a stock market index for species. The index is comprised of thousands of population time-series for vertebrate species from locations around the world; the trends from these populations are averaged to produce terrestrial, freshwater, and marine indices, which are further aggregated to a global LPI. The 2022 global LPI shows a decline of 69% between 1970 and 2018 globally^112,192^. This is an average trend based on time-series data from 31,821 populations of 5230 species of mammals, birds, reptiles, amphibians, and fish.

The LPI database (LPD) can include population data for any species for which time-series population data could be found, regardless of threat status, or whether they show increasing or declining trends. These population time-series are sourced from scientific papers, online databases, government, and expert led published reports. They can be searched and downloaded from the project website along with more technical information on the LPI (http://livingplanetindex.org/).

## The origins and development of the living planet index

The Living Planet Index was conceived in 1997 by the World Wildlife Fund for Nature (WWF International). The primary aim was to “*develop a measure of the changing state of the world’s biodiversity over time*”^18^ using aggregate population trends for a large sample of species from across the world. As very little data were available on plants, fungi or invertebrate species, the pragmatic approach was taken to restrict the initial LPI taxonomically to vertebrates. There was also geographic unevenness in the distribution of the available data: long-term monitoring studies dating back decades were located mainly in Europe and North America. To address the biases in data coverage, a benchmark of 1970 was set, and the data were divided up into three broad biomes – terrestrial, freshwater and marine – and then further into regional groupings. The source data and LPI outputs were at first collaboratively managed by WWF and the World Conservation Monitoring Centre (now UN Environment Programme WCMC) for use within WWF’s flagship publication, the Living Planet Report (LPR). First published in 1998, the LPR used the initial iteration of the LPI as a communications tool to convey biodiversity trends into a singular message on the health of the planet for a broad audience, alongside measures of humanity’s impact on the planet^1^. Calculated as -32% on average between 1970 and 1995 (Loh, et al.^1^), the downward trend of the LPI was already apparent.

In the early 2000’s, as the LPI dataset and methods were developed further^18^, their potential for use in advocacy, research, and as an indicator for monitoring biodiversity were recognised more widely. In 2002, the Parties to the CBD committed to achieve a significant reduction in the rate of biodiversity loss at the global, regional and national level by 2010 and required a framework of biodiversity indicators to monitor their progress^19^. The first national LPI, the ‘Living Uganda Index’, was published with the National Biodiversity Data Bank recording scheme at Makerere University, Uganda in 2004^20,21^ and was presented as a case study for country-level applications of species population indices at CBD COP 7^22^. A Discussion Meeting held at the Royal Society in 2004 brought together leading academic and NGO researchers working on biodiversity indicators, and the resulting papers, including one on the LPI, were published in a special issue of Philosophical Transactions B^23^. This meeting laid much of the groundwork for subsequent indicator development in the context of the CBD and other international biodiversity monitoring processes^24^. In 2005, the Convention’s scientific advisory body adopted the LPI metric as part of a suite of biodiversity indicators, deployed to monitor progress towards that target^25^. In 2010, the CBD Parties agreed a further set of biodiversity targets, the Aichi Targets, for the period 2011 to 2020^3^ and the LPI was identified as an indicator for monitoring progress towards several of these.

To strengthen the LPI’s scientific foundations and improve its capacity as an indicator for tracking progress towards international biodiversity policy targets, an in-depth peer-reviewed paper on the methodology was published^18^ and the current partnership between WWF and the Zoological Society of London (ZSL) was subsequently formed in 2006. Since then, two updates to the methodology behind the global index have been published^26,27^ and the research potential of the LPI data has expanded by incorporating metadata on ecology, geography, threats and management into the database, the core data of which were made openly accessible online in 2013 (18% of the data set is not available due to a confidentiality clause in the data sharing agreement, often for rare or threatened species – see Challenges and opportunities).

## Applications of the LPI

Here we provide an overview of the uses of the different LPI project elements (see Box 1) and outputs, grouped into three themes: public engagement and advocacy, its use in policy and, as a tool for research.

From its inception, the LPI was seen as a powerful tool and WWF communications found that it resonated with the public better than any other conservation messages at that time. The LPI helps to set the scene for the state of global biodiversity by conveying a complex topic as a singular takeaway message for a broad audience. The key conduit for the global LPI has been as the headline biodiversity indicator within the LPR. The LPR is an open access, biennial publication of the latest research and insights into global biodiversity trends, the human drivers behind them, and proposed solutions to halt biodiversity loss and “bend the curve”^9^ back towards restoration. Its widespread distribution and WWF’s communications expertise have provided a regular global media platform, emphasizing opportunities for awareness raising and advocacy regarding the biodiversity crisis. The 13^th^ edition, published in 2020, was translated into 16 languages and circulated around the world, with over 290 million social media views and 3560 mentions from monitored global news outlets within the first month of its launch^28^. The consistent use and media exposure within the LPR has accorded the LPI with familiarity within the public realm (see Challenges and Opportunities). An analysis of online posts and articles (in English) containing the LPR 2020’s keywords or hashtags showed that 51% mentioned the 2020 global LPI statistic^28^. Apart from global LPI figures, analysis of subset indices such as those featured in the LPR 2020 (LPI by The Intergovernmental Science-Policy Platform on Biodiversity and Ecosystem Services (IPBES) regions, taxonomic focus (e.g., reptiles) and ecological biome (e.g., forests and freshwater)) have been used to draw focus towards trends within different species groups^4,29,30^.

Both the underlying data in the LPI and the global results have been used in several educational formats, in schools and higher education. As part of the LPR 2020 outreach campaign, a youth edition including the LPI trends was prepared^31^ and adapted by WWF country offices to enable young people to learn from the report’s key messages and promote engagement of schools globally in biodiversity issues.

Nature documentaries provide another medium for large-scale biodiversity outreach^32^. The 2019 Netflix series “Our Planet,” narrated by Sir David Attenborough, used the global LPI statistic from LPR 2018 to set the scene for its narrative alongside other headline biodiversity indicators and, within the first month of the launch, was viewed by 45 million accounts across the world^33^.

National scale LPI analysis and LPRs such as those undertaken by WWF offices in Belgium^34^, the Netherlands^35^ and Canada^36^, and regional approaches like the 2013 and 2022 editions of the “Wildlife Comeback in Europe” report^37,38^ have used LPI figures to illustrate species trends and raise public awareness to what is happening to status and trends of the biodiversity on their doorstep. The 2013 edition of the Wildlife Comeback report reached 138 million people across Europe and worldwide^39^.

Regarding the use of the LPI project within policy, analyses of the LPI dataset and trends within a geopolitical, ecological or taxonomic focus have been used to provide evidence of biodiversity change for policymakers, fed into policy and target development, and monitored progress towards those targets. The LPI is part of a suite of biodiversity indicators adopted by the CBD, measuring trends in relative abundance of vertebrates and previously deployed to monitor progress towards the 2010 Biodiversity Target^19^, subsequent 2020 Aichi targets^3^, and now is a component-level indicator for Goals A and B and Targets 4, 5 and 9 of the K-M GBF^12,40,41^. As a measure of population trends compiled at annual intervals, the LPI is sensitive enough to detect annual changes, which is of value for informing policy^15^ and evaluating the impact of conservation interventions^19,42^.

ZSL and WWF joined the Biodiversity Indicators Partnership (BIP) in 2007 to further develop the LPI and make it available for use under the CBD strategic plan. This resulted in the use of the LPI as evidence of biodiversity decline in international policy documents (Table 1): global and regional assessments (Millennium Ecosystem Assessment (2005)^43^, IPBES global, regional and thematic assessments^6,44–47^ and successive updates of UN Global Environment Outlook^25,48–51^ and UN Global Biodiversity Outlook^52–55^) as well as thematic assessments (Ramsar Convention on Wetlands, (2018)^56^, Mediterranean Wetlands Outlooks (2012 and 2018)^57,58^, the Convention on Migratory Species reports (CMS) (2008 and in 2019)^59,60^ and Arctic Biodiversity Assessment (2013)^61^). More recently, the global and regional indices were used to illustrate the state of nature and how this varies geographically as part of the evidence base for the Dasgupta review, an independent report on the economics of biodiversity^62^.

**Table 1:** Selected applications of the LPI data and or method and the corresponding and suggested uses for tracking global conventions on biodiversity, sustainable development, and other multilateral environmental agreements (MEAs).

Application of the LPI	Corresponding biodiversity and sustainable development targets and other multilateral environmental agreements (MEAs)		
Disaggregation and reference	CBD GBF	SDG	MEAs
Sub national			
LPI-Cat, State of Nature in Catalonia 2020 report for Catalunya, Spain^166^	Goal A, Target 4	Target 14 and 15	IPBES
Indice Région Vivante (IRV), province of Provence-Alpes-Côte d’Azur, France^66^	Goal A, Target 4	Target 14 and 15	IPBES
Indice Région Vivante (IRV), bird indicator for the province of Franche-Comté, France^167^	Goal A, Target 4	Target 15	CMS and IPBES
National			
Living Uganda Index (LUI), Uganda^21,168–171^	Goal A, Target 4	Target 15	IPBES
Living Planet Index or Naturindeks for Norge, Norway^172^	Goal A, Target 4	Target 14 and 15	IPBES
Canadian Species Index (CSI), one of a suite of Canadian Environmental Sustainability Indicators, Canada^69,173^. The Canadian Living Planet Index (C-LPI)^36^	Goal A, Target 4	Target 14 and 15	IPBES
Living Planet Index Netherlands, the Netherlands^123,174^	Goal A, Target 4	Target 14 and 15	IPBES
Living Planet Index, China^175,176^	Goal A, Target 4	Target 15	IPBES
Belgian Living Planet Index, Belgium^34^	Goal A, Target 4	Target 14 and 15	IPBES
Threatened Species Index (TSX), for birds, Australia^67^	Goal A, Target 4	Target 15	IPBES
The Austrian Living Planet Index, Austria^177^	Goal A, Target 4	Target 15	IPBES
Regional			
Arctic Species Trend Index (ASTI) for vertebrates across the Arctic^61,178,179^	Goal A, Target 4	Target 14 and 15	IPBES and RAMSAR
ASTI for Arctic marine mammals, birds and fish^180^	Goal A, Target 4	Target 14	IPBES
Arctic Migratory Birds Index^181^	Goal A, Target 3	Target 14 and 15	CMS and IPBES
Mediterranean wetlands Living Planet Index^57,58,182^	Goal A, Target 4	Target 6	IPBES and RAMSAR
European marine vertebrates Living Planet Index, European Environment Agency (EEA)^183^	Goal A, Target 10	Target 14	IPBES
Ecological			
Living Planet Index for global estuarine systems^184^	Goal A, Target 10	Target 6, 14 and 15	IPBES and RAMSAR
Living Planet Index for migratory species^60,82^	Goal A, Target 3 and 5	Target 14 and 15	CMS and IPBES
Living Planet Index by marine, freshwater and terrestrial biomes^56,185^	Goal A and B, Target 3, 9 and 10	Target 6	IPBES and RAMSAR
Living Planet Index for Reptiles^84^	Goal A	Target 14 and 15	IPBES
Living Planet Index for freshwater megafauna^83^	Goal A, Target 3 and 5	Target 6 and 15	CMS, IPBES and RAMSAR
Living Planet Index for migratory freshwater fish^186^	Target 3, 5 and 10	Target 6 and 15	CMS, IPBES and RAMSAR
Forest Specialists Index^81^	Goal A and B, Target 2 and 10	Target 6 and 15	IPBES
Conservation management and species utilisation			
Protected areas^78,85^	Goal A and B, Target 2, 3 and 4	Target 15	
Impacts of conservation management on species^42^ and threatened species^36,86^	Goal A and B, Targets 2 and 4	Target 15	
Living Planet Index for recovering populations of European mammals and birds^37^	Goal A, Target 2, 5, 9 and 10	Target 15	IPBES
Living Planet Index for utilized species^88,187^	Goal B component indicator, Target 5 and 9 component indicator*	Target 8, 12, 14 and 15	CITES and IPBES
Trends in target and bycatch species (oceanic sharks and rays)^188^	Goal B, Target 5 and 9, Complementary indicator	Target 14	IPBES
Other influences of the LPI			
Index of Linguistic Diversity^96,189^	Goal B, complementary indicator*	Target 1 and 16	IPBES
The Wetland Extent Trends Index^94,95^	Goal A, complementary indicator*	Target 6	IPBES and RAMSAR
Sustainability Policy Transparency Toolkit (SPOTT) Index^97^	Target 15 Complementary indicator	Target 12	
The Species Awareness Index (SAI)^98^	Target 21 Complementary indicator		

An asterisk and bold text denote an indicator formally included within the proposed Kunming-Montreal global biodiversity framework. Sourced from: *UNEP* (United Nations Environment Programme)^12,41^, *UN* (United Nations)^13^^,^ *UNEP*-*WCMC* (UN Environment Programme World Conservation Monitoring Centre)^190^, *UNEP* (United Nations Environment Programme)^191^.

LPIs have been used as a scientific basis and in their scene setting capacity, to influence policy development when advocating for transformative change and setting ambitious biodiversity targets^8,9^. The global LPI statistic has featured in high-level biodiversity discussions, for example within Volkan Bozkir’s (President of the UN General Assembly) speech to heads of state at the 75^th^ UN Summit on Biodiversity in 2020 and within UK parliament in 2016 to support an Early Day Motion on Global Biodiversity^63^.

The LPI dataset and guidance on applying the method at a sub-global scale^64^ have allowed for regional, national and in some areas, sub-national scale analysis (Table 1). This ‘scalability’ is a key requirement for indicators to be effective at tracking progress of signatory parties towards larger intergovernmental targets^64,65^. CBD parties, for example, can develop national LPIs to fulfill part of their reporting requirements within their National Biodiversity Strategy and Action Plans (NBSAP)^2^. Several members, including the Netherlands, Uganda, Canada, and China have provided LPI analysis of species trends within their NBSAP reports. In France, this process has been scaled down even further and provinces such as Provence-Alpes-Côte d’Azur have used LPI analysis to track progress towards their National Biodiversity Strategy^66^. In Australia, a new application of the LPI method focussed on threatened species to monitor their national progress towards Aichi Target 12 (extinction prevented)^67^.

Aside from tracking CBD commitments, nations have adapted the LPI method and applied it to suit their state biodiversity indicator needs such as the “Canadian Species Index,” developed by ZSL in partnership with Environment and Climate Change Canada (ECCC)^68,69^. The package in the programming language “R” for calculating the LPI (rlpi), is freely available via GitHub^70^, and has been used by collaborators from around the world to produce their own regional and national indices e.g. national and scientific agencies within Brazil use it within a national bird and mammal monitoring programme^71^. However, application of the LPI at the national level (Table 1) has largely remained restricted to a few, largely high income, countries. A recent study of data availability for priority species in East Africa found that, although of the greatest importance for conservation projects, data on species abundance was the hardest to access^72^. This exemplifies the deficit in capacity and resources available for collecting and analysing species data at the national scale.

**Fig. 1:**
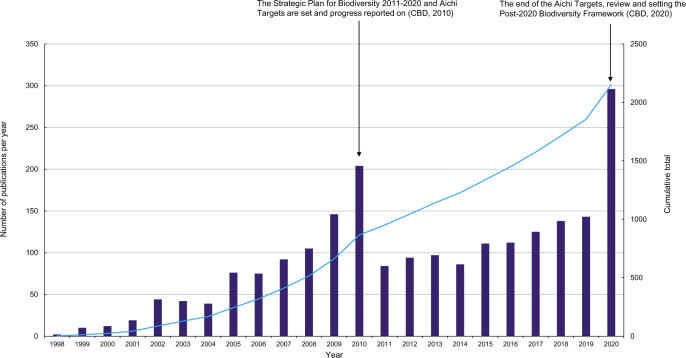
The number of publications per year citing the Living Planet Index between 1998 and 2020. The secondary Y-axis shows the cumulative total of publications. These 2152 citations are from academic and grey literature in English and non-English languages between the years 1998 and 2020 (as of 18th of January 2021). See Supplementary Methods 1 and Supplementary Table 1 for details on the methods and Supplementary Note 1, Supplementary Figs. 1–3 and Supplementary Tables 2–5 for further exploration of the citation data, and Supplementary Table 6 and Supplementary Fig. 4 for key LPI papers’ citation impact.

Examining the LPI project as a tool for research, the LPI methods, dataset and metrics have been used either individually or in unison for numerous research projects around the world (Table 1 and Fig. 1). Within a random sample of 341 citations containing the term “Living Planet Index,” 90% of author and document affiliation was classed as research (academic institution or university); of the outputs themselves, 53% were within academic journals (Supplementary Methods 1, Supplementary Note 1 and Supplementary Tables 4, 5).

The Living Planet Database (LPD) (except for about 18% of the data marked as confidential – see the dataset section under Challenges and opportunities) has been publicly available since 2013 when the LPI website was created to facilitate viewing and downloading the data. Prior to this, subsets of the database were shared upon request. The LPD is now the largest repository of vertebrate population trend data (containing over 38,000 populations of more than 5,200 species at the time of writing), adding to a wealth of available biodiversity data for species occurrence (GBIF^73^), species extinction risk (IUCN Red List^74^) and ecological community data (PREDICTS^75^, BioTIME^76^). To date, www.livingplanetindex.org has had over 6,000 registered users from 145 countries around the world.

Within the LPD, the population and ancillary data (Supplementary Fig. 5) have facilitated a wide range of research topics (Table 1). In particular, the threat and management data at population-level allows for more fine-grained analysis compared with using species-level data. Recent applications of the data include: measuring the effectiveness of protected areas;^77–79^ evaluating the correlates of abundance trends in subsets of species such as mammals, reptiles, forest specialists, freshwater megafauna and migratory species;^80–84^ the nature of population dynamics in response to threats or management;^85–89^ the effects of land use and climate on species^90^ and exploring linkages between human development variables and wildlife population trends^91^.

The LPD has been incorporated into an open access repository at the University of Edinburgh, dedicated to providing free online courses in statistics for ecology and environmental scientists^92^. In a more informal setting, LPI data have been used to present challenges for data visualisation or analysis as part of Hackathons, one of which led to the development of a tool to automatically identify papers containing abundance data^93^.

The framework used to calculate the LPI has been applied to produce other metrics and not just for biodiversity. Conceptually, relative change, as calculated by the geometric mean, can be applied to other units of measurement that have been collected consistently over time. Using the code for calculating the LPI, new indicators have been developed for wetland areas^94,95^, linguistic diversity^96^, monitoring environmental, social and governance transparency in palm oil production^97^ and biodiversity awareness^98^. The first two of these are part of the ongoing suite of indicators for the CBD (Table 1).

## Challenges and opportunities

Along with other high-profile biodiversity indicators and reports^99,100^, the underlying data, methods, and interpretation and communication of the LPI have repeatedly come under scrutiny, which has been a positive catalyst for new research, collaborations and ameliorations on the scientific rigour of the index. Here we provide an outline of the challenges faced by the LPI and aim to provide clarity on common misconceptions that have arisen within recent years.

**Fig. 2:**
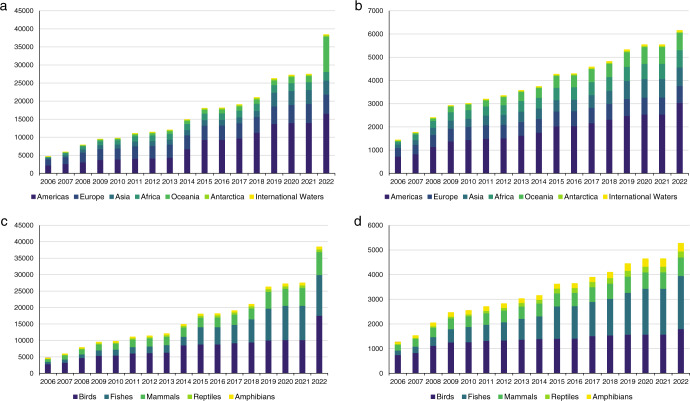
Growth in number of populations and species in the Living Planet Database (LPD) by region and taxa. **a**–**d** The cumulative number of new populations (**a**) and species (**b**) entered by region, and the cumulative number of populations (**c**) and species (**d**) entered by taxon. Please note 2b adds up to more than the individual number of species as some species occur in more than one region. See Supplementary Methods 2 for details how the figure was derived and Supplementary Fig. 6 for an overview.

One of the strengths of the LPD (the dataset underpinning the LPI) is that it is not static: data are continually added and updated to provide the most complete and accurate picture possible of trends in relative abundance (Fig. 2). To ensure data are comparable, only species-level time-series which fulfil the following criteria are added: they are a measure of population abundance (or proxy, such as number of breeding pairs), with two or more years of data, collected within a specified geographic location under consistent methods (or explicitly corrected for)^26^. Supplementary metadata (Supplementary Fig. 5) are continually updated for both new, and existing time-series, adding a further step in the data extraction process^26^. The rigorous evaluation of data sources and data extraction not only limits the amount of applicable data that can be included, but it is also time consuming and labour intensive, and affects the volume of data that can be processed for each update. Storing these data in suitable infrastructure and the financial support required to maintain it are a further limitation common to other biodiversity databases^101^. The costs of running the entire project can be complex to calculate as the source data are often already published and there are many stakeholders including researchers and policymakers to consider.

Long-term, abundance studies at a species population level are a limited resource in themselves, particularly for highly speciose taxa such as invertebrates and plants which have not been included in the LPD to date (see The future). Studies that include population data may not have been designed for long-term population monitoring but to assess population size and so their methods and survey effort might change with advances in population estimate approaches (e.g., revised Orangutan estimates in Sabah^102^). This can render data incompatible for inclusion in the LPD. This issue is amplified for regions and taxa which are recognised as underrepresented within the dataset such as tropical regions and fish, reptiles and amphibians (Supplementary Tables 7, 8)^27,103^.

Consequently, the composition of the LPD is likely to reflect biases inherent in species monitoring schemes which tend to favour certain taxa (e.g., birds) or regions (e.g., high-income countries)^27,104,105^. This is a challenge shared by biodiversity indicators and databases in general^99,106^. In addition, attempts to source data from grey literature or offline databases is often dependent on the time and expertise available from researchers and field contacts within chronically neglected and underfunded areas^107^. To counteract bias in the resulting LPI, two approaches are taken. At the data inputting stage, a gap analysis of the taxonomic and geographic representation of the LPD is used to prioritise taxa and regions for targeted data searches (Supplementary Fig. 7). However, focussed searches are not always fruitful: within the 2020 LPI, only 4 populations of African amphibians were included despite targeted efforts^108^. The second step for overcoming bias in the LPI is in the adoption of the diversity-weighted method (see details on the method below).

Language is a further constraint to collating representative data for the LPI and can exacerbate existing geographic biases^109^. The dominance of English-language data sources is partly a reflection of the LPI project being hosted in an English-speaking country but also of English as a globally used language for science^110^. However, over a third of biodiversity documents from a single year were published in languages other than English^111^, so there are likely to be data that have not been captured because language barriers have not yet been adequately addressed. Broadening the number of languages used for compiling data could help to improve the development of national LPIs. The latest Living Planet Report reported on the efforts by Brazilian researchers to boost the national data set in the LPI through literature searches in Portuguese: over 2500 populations and 575 new species were added within a few months^112^.

Collating and storing a continually increasing repository of LPI data, that aligns with FAIR (Findable, Accessible, Interoperable, and Reusable) Data Principles, requires ongoing investment in the data infrastructure and management^101,113^. Coupled with this is the importance of promoting data sharing in a way that alleviates concerns over data ownership and provides appropriate credit to data providers. Unless a system is in place whereby data providers maintain ownership and control of their data, there is likely to be a barrier to mobilising data. Currently, 18% of the data in the Living Planet Database are marked as confidential, meaning they are not available to third parties because the species is rare or threatened, the data are being used in another publication underway, or further data sharing is limited by other agreements or contracts. This compromises the transparency and reproducibility of the LPI as well as resulting in a smaller public dataset available for research. Whilst it remains important to respect the protection of sensitive data, there are some remedial actions that could be taken to maximise the data available. Firstly, data could be anonymised where possible, for example by removing the identity of the species, location and even country. This could allow a greater proportion of either the raw data or annual trend values to be used. Further, ongoing efforts could be made to encourage data providers to release older data sets from the confidentiality clause, as they may have served their original purpose and could be made readily accessible.

The key methodological challenges for the LPI project are to generate a robust indicator of biodiversity and to model the time-series data in the LPD, which vary in length and scale, in a way that allows exploration of underlying patterns in population trends. A further challenge that underpins both issues, is addressing the taxonomic and geographic gaps in the underlying data (Supplementary Tables 7, 8).

The basic formula for calculating the LPI has remained largely unchanged: each logged population trend is averaged within a single species and the species trends are aggregated to produce a single index^18^. This aggregation is produced using a geometric mean, an approach used to generate other indices of relative abundance from species abundance data^114–116^. Further levels of aggregation are often used for global, national, and local contexts (see Supplementary Fig. 7 for the global example).

A challenge in the use of a geometric mean of abundance for the calculation of indicators is that it can be sensitive to outliers in the data which may impact the precision of the long-term trend if not addressed^117–119^. While this method is still considered to be a more suitable and sensitive metric to assess changes in biodiversity^120,121^, understanding the impact of outliers is important. To tackle this, each new iteration of the global LPI analysis includes sensitivity tests on the influence of single species on the trends and of the effect of short time-series on the LPI, as these are more commonly associated with highly variable or extreme trends^108^. These tests are published in the supplementary information, blog or website for transparency and to demonstrate the robustness of any index^108^.

Another property of the geometric mean as used in the LPI is that it measures relative abundance or average rates of change, not trends in the absolute abundance of individual animals^122^. Whilst this has presented challenges in the communication of the results Puurtinen et al.^122^, (discussed below), the use of a geometric mean may lend the LPI to being a sensitive indicator of species recovery as it does not tend to be dominated by trends in abundant species, which are often stable or increasing.

The modelling of the time-series data in the LPD has been periodically improved. In early iterations of the LPI, the chain method was implemented, which involved linearly interpolating the rate of change between 5-year intervals, (following Loh, et al.^18^). As this approach was sensitive to abrupt changes in population trends, generalised additive modelling (GAM) was adopted to better capture long-term nonlinear trends in populations^26^. National variations of modelling have been tailored to the type of species monitoring data in the country in question^123^, for example the use of linear regression for short-term trends in the Canadian Species Index^68^.

More recently, Bayesian approaches such as state-space models have been applied to model the population time-series whilst incorporating observation error into the estimation of trends^124^, which the GAM framework does not account for. This has allowed for new ways of analysing the LPD, which lend themselves to uncovering the correlates of vertebrate population trends^125^ and the taxonomic and geographic patterns of population trends globally^119^.

A significant challenge remains in tackling the underrepresentation in the LPI database of particular taxa and regions in the LPD. An adaptation to the LPI, the diversity-weighted approach, was developed to mitigate the impacts of this bias on the index and subsequently adopted for calculating global and regionals LPIs^27^. This method places greater weight on species trends from regions and taxa that are more species-rich but tend to be disproportionately under-represented in the LPD e.g., the Neotropics. This provides a more representative picture of global vertebrate trends in lieu of a more complete dataset. One drawback is that weight is often placed on species and regions with the lowest data availability so if the sample of data from a region is not representative, this could cause an over- or under- estimation of trends. As noted above, efforts are also underway to address gaps in the data set through targeted data collection and to develop models to predict trends in locations and for taxa which are data deficient, as has been done for extinction risk^126^.

Key attributes of biodiversity indicators are that they should be simplified and easily understood^116^. The LPI was developed with these criteria in mind and, by aggregating trends from different ecological realms and geographic regions, it can provide a useful overview and communication tool for broad audiences. However, the index has been critiqued as oversimplifying the state of biodiversity^127^ and masking important trends^119^. Furthermore, it has been argued that the LPI is not measuring what it should, or what it claims to (i.e., abundance), and that it is hard to interpret and appears not to behave as expected (e.g. when the absolute number of animals increases within a group of populations, the index may still show a decline)^122^. The assertion that the LPI does not measure abundance is valid but, as explained here, the LPI was not developed to measure abundance but rather change in relative abundance. There are scientific arguments for the utility of a relative abundance indicator to monitor biodiversity^115,120^ and other examples are in use in policy^118,128^. However, it is true that this nuance is likely to be glossed over by a broader audience, contributing to confusion over what the LPI shows^122^. This argues for placing more emphasis on improving the explanation of precisely what the LPI is measuring and providing clear guidance to ensure it is correctly interpreted.

The difficulties underlying the communication of biodiversity indicators are not unique to the LPI and present a challenge to the scientific community to try to overcome. For example, global statistics of changes in forests have been reported on using the Global Forest Watch dataset as their basis^129^. However, uncertainty within the underlying dataset around detection of forest cover changes can underrepresent loss^130,131^. Arguably, there is need for a balance between providing a simple, clear message about global biodiversity trends whilst supporting it with more in-depth analysis^100^. To explore and uncover this variation, disaggregations of the LPI have been developed (Table 1), for example, for forest specialists^81^.

The limited availability of quality, ecological data prior to the 1970s is a common limitation to many biodiversity indicators^106,132^. The LPI is benchmarked at a temporal baseline of 1970, and this raises the importance of interpreting the index in context, as geopolitical regions have been impacted by anthropogenic pressure at different points in time and varying intensity. In Europe, for example, a significant amount of habitat destruction and overexploitation of some species had occurred prior to the 1970s and therefore the LPI baseline is set at a significantly depleted reference point^37,133,134^. The year chosen as a baseline can affect the interpretation of the state of biodiversity in a particular region^135^. Without taking this into consideration, it is possible to underestimate the gravity of the decline in biodiversity or overestimate a recovery within any given landscape.

It can be challenging to ensure that a nuanced indicator such as the LPI is correctly interpreted across all audiences, especially when reported on across the globe. Communications around biodiversity indicators and biodiversity loss have often centred on species, and species extinctions, respectively, rather than attempting to explain the multi-faceted nature of biodiversity change and how we measure it^100,136^. Miscommunication and oversimplification of biodiversity and biodiversity loss, or decline, across the science-society and science-policy interface, are challenges shared by biodiversity indicators in general^100^ and will take a collaborative-minded approach driven by the scientific community to resolve. The importance of how language is used to communicate trends has been illustrated recently with the publicity for the Living Planet Report: the impact of substituting a single word for another in press and media communications, namely “loss” vs “decline”, potentially exacerbated the misinterpretation of the global LPI statistic. A negative trend in the LPI depicts a relative decline in population sizes, on average, since 1970. The use of the word “loss” in some media articles can imply that a negative LPI trend is analogous with the disappearance of populations and even the extinctions of species, which can prove challenging to correct. Media headlines have referred to large percentages of populations being “wiped out”^137^, which could mislead the public about the severity of biodiversity decline and, it has been argued, such negative statements about environmental issues may be counterproductive in trying to stimulate action^138^.

Efforts to minimise misinterpretation are made with each iteration of the LPI, by engaging with journalists directly through press briefings and providing background information to communications teams on the LPI and the part it plays within the global biodiversity indicator toolkit. This is bolstered by publicising the supporting information available in technical supplements to the LPR^108^, providing visualisations and tools to allow exploration of the data and better understanding through websites (https://www.livingplanetindex.org/stats) and blogs^139^. These efforts and a consistent use of key terms could also help to reinforce the LPI as a measure of “relative abundance” rather than “abundance” to avoid misinterpretations^122^. There has been an uptake in the use of the LPR 2020 technical supplement in recent publications and blogs exploring the LPI^140,141^. The analogy of a FTSE index for biodiversity is most commonly used to describe the LPI, but a focus in the future should be on finding other ways to communicate the index that mitigates the use of dramatic narratives, whilst retaining the simple message of the LPI that can be broadly understood. Lessons could be taken from the communication styles used for reporting other biodiversity indicators. For example, results from the Biodiversity Intactness Index report changes in the index itself rather than what it represents (https://www.nhm.ac.uk/our-science/data/biodiversity-indicators/biodiversity-intactness-index-data). Similarly, the LPI results could be reported as the increase or decline in the *index* between two time points rather than referring to trends in populations or abundance, which may be misconstrued. This could be followed by an explanatory sentence highlighting the importance distinction in the meaning of the index, e.g., “The Living Planet index has declined on average by 69% since 1970.” This describes the average change in *relative* abundance over time, not change in overall abundance.” The UK State of Nature report uses phrasing that also incorporates what the indicator is a measure of^142^. This could be echoed in the reporting of the LPI by using a phrase such as, “the Living Planet Index of changes in relative abundance of 5230 vertebrate species has declined by 69% since 1970.”

## The future

The LPI project has grown significantly over the last 25 years and provides an important dataset to communicate the trends in vertebrate populations and investigate the factors that influence them. We identify four key priorities for the immediate future.

1. Increasing representation in the LPI. The composition of the LPI needs to be improved, crucially by increasing the taxonomic and geographic representation of the data, particularly for aquatic species. Incorporating invertebrate and plant species into the LPI could be challenging given either the paucity of monitoring compared to some vertebrate groups^143^ or the difference in types of data used for measuring abundance for these taxa. However, efforts to accommodate a wider taxonomic diversity are key if the LPI is to capture and communicate trends in global biodiversity and in turn, will provide a more powerful dataset for macro-ecological research. Global initiatives within the research community such as the Status of Insects project^144^ and the State of the World’s Plants and Fungi reports^145^ may provide opportunities to harness data and incorporate invertebrate, plant and fungi species into the LPI. The sampled approach to the Red List Index was employed to broaden the taxonomic coverage of this biodiversity indicator^146–148^, and a similar strategy for the LPI may be a pragmatic way of tackling the same issue.
  Many national LPIs have already been developed, and maintaining this focus on increasing the representation of species within countries will provide nations with a tool to track progress towards future CBD and SDG targets. Indicators also need to be ecologically relevant^116^, so ensuring that different functional attributes of species within an ecosystem are reflected will be the focus of new research. These developments in the data set could also be realised through the use of emerging techniques to incorporate unstructured data, such as that collected through citizen science initiatives^149–151^, and capitalising on growing technology for monitoring biodiversity such as eDNA, satellite monitoring and AI-assisted counting of species, provided they can be transformed into usable metrics of abundance. Whilst these approaches will primarily enrich the representation of data in the future, the science behind linking and predicting biodiversity trends to environmental changes and drivers is continually growing^8,152^ and may offer opportunities to hindcast species trends to an appropriate baseline using climate and land use data, making them usable in long-term indicators^153^.
2. Streamlining data collation and data access. Sourcing and extracting data continue to be significant bottlenecks for the development of the LPD. Data searches can be automated to some degree using predictive models based upon titles and abstracts^93^, but extracting data automatically remains a challenge. Working with publishers, data holders, government institutions and research funding bodies to automate the process of identifying and extracting data from articles would be beneficial particularly if a standardised workflow is developed (e.g., Cardoso, et al.^154^ and Hochkirch, et al.^143^), and systematic review tools may advance data collation in a community-driven way^155^. To address language barriers, which in turn could help to fill taxonomic and regional data gaps^156^, a protocol for conducting data searches in multiple languages is under development and has been applied in a pilot project in Brazil^112^. This should be part of a broader strategy to build a sustainable data network for the LPI, which provides accessibility to a global database (both for data download and upload, e.g., from new national LPI datasets) whilst retaining data quality and ownership, and assuring appropriate credit to data gatherers and providers. It is also important that the LPD is made as accessible as possible, both through simple, downloadable, tidy data formats^157^ and the development of Application Programming Interfaces (API) to allow the data to interoperate with other resources such as the IUCN Red List^74^, Protected Planet^158^ and GBIF^73^.
3. Better models to link population trends with drivers. The LPI continues to highlight that global biodiversity is in trouble and understanding (and predicting) which regions and species are likely to decline most in the future is useful. As such, models to better predict wildlife abundance trends for species and regions where we have poorer data is critical. Understanding the quality and utility of these models will allow us to make concrete and valuable predictions. The varied response of some populations to their changing environment highlights an important question – are some populations useful ‘canaries’ of pending ecosystem collapse and how might we best identify them?
  Models that combine LPI data with drivers such as land-use and climate-change data have demonstrated that both are important drivers of population trends^90^. Developing these models further allows us to make predictions about how biodiversity might change under future scenarios and management interventions^8^, highlighting one evolving use of biodiversity datasets like the LPD.
  Whilst incorporating data on drivers from other global data sets can inform explanatory analysis for species trend data^90^, population-scale information can also provide a powerful set of variables, for example in understanding the effect of different direct drivers^88^ or to pave the way for counterfactual analysis of different management types (e.g. Jellesmark, et al.^159^). However, the current coding for threats and conservation action in the LPD lacks alignment with established frameworks^160^, so transferring the ancillary information into these classification schemes and maintaining the recording of population drivers will improve the utility of models and ground-truthing of broad scale datasets in the future.
4. Increasing the utility of the LPI for policy. From a policy perspective, an emphasis on developing LPIs at the national level is needed to expand its use as a communication and reporting tool. With reporting requirements at a national level for the SDGs and the CBD, national LPIs would serve a dual purpose of providing countries with a sensitive indicator for reporting while boosting data representation for the global index. To achieve this, the barriers to national use of the LPI need to be addressed: improve access to resources and technology to process and analyse data^72,161^, address uncertainty about the suitability of the LPI data and method along with clear guidelines for national use^161^, and broader use of languages to mobilise national data sets^156^. Although the LPI results are widely disseminated, the index and database are not always known about by practitioners^72^, which emphasises the importance of improving data access mentioned above. Disaggregations of the LPI on themes such as use, trade, migration and wetlands should continue to be developed, so that these are available for reporting against other multilateral environmental agreements such as the Ramsar Convention on Wetlands, CITES and the CMS.
  The LPI performed well in an evaluation of biodiversity indicators using decision science^17^, although gaps were identified in the practice of regular tests of the index and in assessing the cost- effectiveness of the LPI relative to other indicators. Creating a better understanding of how the LPI complements the growing suite of biodiversity indicators such as the Red List Index^162^ and the Biodiversity Intactness Index^163^, and clearly presenting these indicators as a package as opposed to alternatives, will be key to developing a clear and consistent narrative of global biodiversity change^14^ and to ensure the suitability of the LPI within any multi-dimensional indicator framework^164,165^.

## Lessons learned

From this review, we have identified lessons learned for the development of global biodiversity indicators and share these below. We urge for open dialogue and investment from within the scientific community to collaborate on solutions within this critical decade.

- Communication. When documenting the challenges for the LPI, communications approaches surfaced as one of the key issues. This is a common challenge not restricted to biodiversity indicators, but it is particularly important given their application and potential impact within policy and public engagement. Tailored approaches for the science-policy and science-public interface may be needed to ensure that results are communicated in a clear way without losing the scientific meaning. Stronger engagement with specialists and teachings from the discipline of science communication could hold vital opportunities for our sector to strike the right balance.
- A diverse data network. Some of the challenges identified in this review may have been mitigated by an initial development of a network of data holders and national or regional indicator producers; the IUCN Red List Index, for example, benefits from being able to call on a network of more than 10,000 experts to conduct Red List assessments. Establishing a network across regions and incorporating non-English language sources from an early point in indicator development could help address bias in geographic representation of the data from the outset. This is particularly key for global indicator development where primary data is used.
- Policy relevance. Whilst the LPI has been used for international policy since 2006, its use at the regional and national scale is less common. One key lesson is to encourage and enable national uptake of indicators both to build country-level data sets and test the method at different scales. This can be done through engagement with national entities to discuss needs, provide training, translate materials and promote the development of regional networks to foster long-term peer support.
- Transparency. Each iteration of the LPI method was peer-reviewed and the database has been publicly available since 2013. However, some of the criticisms of the indicator, and some misunderstandings, may have been avoided. For example, by making the results of sensitivity tests visibly available alongside the publication of the results and providing the specific dataset behind each global LPI available to enable reproducibility. The provenance of the data behind an indicator and how data are selected should also be clearly described, both to increase understanding of the index calculation but also to illustrate to data holders how their data is being used. When it comes to making data available for scrutiny, indicators should strive to follow the FAIR data principles and work to enable equitable data use (e.g. using APIs, publishing data standards and data entry protocol).

The LPI has evolved from a simple communications tool to a large and growing database, policy tool and foundation for research. The open-access dataset and method are globally important resources for the scientific community and beyond, but improvements are still needed to enhance the representation of biodiversity in the underlying data and produce clear and meaningful outputs. Collaboration and engagement within the fields of science, policy, conservation and communication - some of which have fuelled much of the development to date - will continue to be important for ensuring the LPI project remains policy relevant and fit for purpose.

## Methods

For Fig. 1 and Fig. 2 see Supplementary Methods 1 and 2 respectively.

## Supplementary information

Supplementary information

## Data Availability

The datasets used during the study are available from the corresponding author on reasonable request.

## References

[1] Loh, J. et al. Living planet report: 1998. (WWF, Gland, Switzerland, 1998).

[2] UN (United Nations). Convention on biological diversity. 5th June 1992. (United Nations Conference on Environment and Development, Rio de Janeiro, 1992).

[3] Tittensor, D. P. et al. A mid-term analysis of progress toward international biodiversity targets. *Science* **346**, 241–244 (2014). 25278504 10.1126/science.1257484

[4] WWF. Living planet report 2020 - Bending the curve of biodiversity loss. (WWF, Gland, Switzerland, 2020).

[5] Diaz, S. et al. Assessing nature’s contributions to people. *Science* **359**, 270–272 (2018). 29348221 10.1126/science.aap8826

[6] IPBES. Global assessment report on biodiversity and ecosystem services of the Intergovernmental Science-Policy Platform on Biodiversity and Ecosystem Services. (IPBES Secretariat, Bonn, Germany, 2019).

[7] Diaz, S. et al. Pervasive human-driven decline of life on Earth points to the need for transformative change. *Science* **366**, 10.1126/science.aax3100 (2019). 10.1126/science.aax3100 31831642

[8] Leclère, D. et al. Bending the curve of terrestrial biodiversity needs an integrated strategy. *Nature* **585**, 551–556 (2020). 32908312 10.1038/s41586-020-2705-y

[9] Mace, G. M. et al. Aiming higher to bend the curve of biodiversity loss. *Nat. Sustainability* **1**, 448–451 (2018).

[10] Xu, H. et al. Ensuring effective implementation of the post-2020 global biodiversity targets. *Nat. Ecol. Evol.* **5**, 411–418 (2021). 33495589 10.1038/s41559-020-01375-y

[11] Chan, S. et al. The global biodiversity framework needs a robust action agenda. *Nat. Ecol. Evol.* **7**, 172–173 (2023). 36443469 10.1038/s41559-022-01953-2

[12] UNEP (United Nations Environment Programme). CBD/COP/DEC/15/5 19 December 2022. ADVANCE UNEDITED: 15/5 Monitoring framework for the Kunming-Montreal Global Biodiversity Framework. Decision adopted by the Conference of the Parties to the Convention on Biological Diversity. (Convention on Biological Diversity (CBD), Montreal, Canada, 2022).

[13] UN (United Nations). Transforming our world: The 2030 agenda for sustainable development. (United Nations (UN), 2015).

[14] Hill, S. L. L. et al. Reconciling Biodiversity Indicators to Guide Understanding and Action. *Conserv. Lett.* **9**, 405–412 (2016).

[15] Jones, J. P. et al. The why, what, and how of global biodiversity indicators beyond the 2010 target. *Conserv. Biol.* **25**, 450–457 (2011). 21083762 10.1111/j.1523-1739.2010.01605.x

[16] Nicholson, E. et al. Making robust policy decisions using global biodiversity indicators. *PLoS ONE* **7**, e41128 (2012). 22815938 10.1371/journal.pone.0041128 PMC3399804

[17] Watermeyer, K. E. et al. Using decision science to evaluate global biodiversity indices. *Conserv. Biol.* **35**, 492–501 (2021). 32557849 10.1111/cobi.13574

[18] Loh, J. et al. The Living Planet Index: Using species population time series to track trends in biodiversity. *Phil. Trans. R. Soc. B* **360**, 289–295 (2005). 15814346 10.1098/rstb.2004.1584 PMC1569448

[19] Butchart, S. H. et al. Global biodiversity: Indicators of recent declines. *Science* **328**, 1164–1168 (2010). 20430971 10.1126/science.1187512

[20] Arinaitwe, H., Pomeroy, D. E. & Tushabe, H. The State of Uganda’s Biodiversity: 2000. 56 (National Biodiversity Data Bank, Makerere University Institute of Environment and Natural Resources, Kampala, Uganda, 2000).

[21] Pomeroy, D. & Tushabe, H. The State of Uganda’s Biodiversity 2004. (National Biodiversity DataBank (NBDB). Makerere University Institute of Environment and Natural Resources (MUIENR), Kampala, Uganda, 2004).

[22] Jenkins, M., Kapos, V. & Loh, J. Rising to the biodiversity challenge. The role of species population trend indices like the Living Planet Index in tracking progress towards global and national biodiversity targets. (World Bank, Washington, DC. USA, 2004).

[23] Balmford, A., Crane, P. R., Green, R. E. & Mace, G. M. Discussion Meeting Issue ‘Beyond extinction rates: monitoring wild nature for the 2010 target’. *Phil. Trans. R. Soc. B* **360**, 219–477 (2005).

[24] UNEP (United Nations Environment Programme). Decision VIII/15: Framework for monitoring implementation of the achievement of the 2010 target and integration of targets into the thematic programmes of work. Adopted at the 8th Conference Of The Parties (COP) to the Convention On Biological Diversity (CBD). (UNEP, Curitiba, Brazil, 2006).

[25] UNEP (United Nations Environment Programme). Report on the eighth meeting of the Conference of the Parties to the Convention on Biological Diversity, CBD. (UNEP, Nairobi, 2006).

[26] Collen, B. et al. Monitoring change in vertebrate abundance: The Living Planet Index. *Conserv. Biol.* **23**, 317–327 (2009). 19040654 10.1111/j.1523-1739.2008.01117.x

[27] McRae, L., Deinet, S. & Freeman, R. The diversity-weighted Living Planet Index: Controlling for taxonomic bias in a global biodiversity indicator. *PLoS ONE* **12**, e0169156 (2017). 28045977 10.1371/journal.pone.0169156 PMC5207715

[28] WWF. Living Planet Report 2020 - Network results. (WWF, Internal report, 2021).

[29] WWF. Living planet Report 2020. Bending the curve of biodiversity loss: a deep dive into climate and biodiversity. (WWF, Gland, Switzerland, 2020).

[30] WWF. Living planet Report 2020. Bending the curve of biodiversity loss: a deep dive into freshwater. (WWF, Gland, Switzerland, 2020).

[31] WWF. Living Planet Report 2020 youth edition: A guide for our future. (WWF, Gland, Switzerland, 2020).

[32] Jones, J. P. G., Thomas‐Walters, L., Rust, N. A., Veríssimo, D. & Januchowski‐Hartley, S. Nature documentaries and saving nature: Reflections on the new Netflix series Our Planet. *People Nat.* **1**, 420–425 (2019).

[33] WWF/tve. Our Planet: Our impact - The first year of the Our Planet Project. (WWF-UK, Woking, UK, 2020).

[34] WWF. Rapport Planète Vivante - La Nature en Belgique. (WWF, Brussels, Belgium, 2020).

[35] Wereld Natuur Fonds. Living Planet Report Nederland. Natuur en landbouw verbonden. (WWF-NL, Zeist, 2020).

[36] WWF-Canada. Living planet report Canada: Wildlife at risk. (World Wildlife Fund Canada, Toronto, Canada, 2020).

[37] Deinet, S. et al. Wildlife comeback in Europe: The recovery of selected mammal and bird species. Final report to Rewilding Europe by ZSL, BirdLife International and the European Bird Census Council. (ZSL, London, UK, 2013).

[38] Ledger, S. E. H. et al. Wildlife Comeback in Europe: Opportunities and challenges for species recovery. Final report to Rewilding Europe by the Zoological Society of London, BirdLife International and the European Bird Census Council., (ZSL, London, UK, 2022).

[39] Rewilding Europe. Annual review 2013. (Rewilding Europe, The Netherlands, 2013).

[40] UNEP (United Nations Environment Programme). in *24th Meeting of the Subsidiary Body On Scientific, Technical And Technological Advice (SBSTTA)* (Convention on Biological Diversity (CBD), 2020).

[41] UNEP (United Nations Environment Programme). CBD/COP/DEC/15/4 19 December 2022. 15/4 Kunming-Montreal Global Biodiversity Framework. Decision adopted by the Conference of the Parties to the Convention on Biological Diversity. (Convention on Biological Diversity (CBD), Montreal, Canada, 2022).

[42] Jellesmark, S. et al. Assessing the global impact of targeted conservation actions on species abundance. Preprint at 10.1101/2022.01.14.476374 (2022).

[43] Millennium Ecosystem Assessment. Ecosystems and Human Well-being: Biodiversity Synthesis. (World Resources Institute, Washington, DC., 2005).

[44] IPBES. The IPBES regional assessment report on biodiversity and ecosystem services for the Americas of the Intergovernmental Science-Policy Platform on Biodiversity and Ecosystem Services. (Intergovernmental Science-Policy Platform on Biodiversity and Ecosystem Services. IPBES Secretariat, Bonn, Germany, 2018).

[45] IPBES. The IPBES regional assessment report on biodiversity and ecosystem services for Europe and Central Asia of the Intergovernmental Science-Policy Platform on Biodiversity and Ecosystem Services. (Intergovernmental Science-Policy Platform on Biodiversity and Ecosystem Services. IPBES Secretariat, Bonn, Germany, 2018).

[46] IPBES. The IPBES regional assessment report on biodiversity and ecosystem services for Africa of the Intergovernmental Science-Policy Platform on Biodiversity and Ecosystem Services. (Intergovernmental Science-Policy Platform on Biodiversity and Ecosystem Services. IPBES Secretariat, Bonn, Germany, 2018).

[47] IPBES. The IPBES regional assessment report on biodiversity and ecosystem services for Asia and the Pacific of the Intergovernmental Science-Policy Platform on Biodiversity and Ecosystem Services. (Intergovernmental Science-Policy Platform on Biodiversity and Ecosystem Services. IPBES Secretariat, Bonn, Germany, 2018).

[48] UNEP (United Nations Environment Programme). Global Environment Outlook 3: Past, present and future perspectives. (United Nations Environment Programme, Nairobi, Kenya, 2002).

[49] UNEP (United Nations Environment Programme). Global Environment Outlook 4: Environment for development. (United Nations Environment Programme, Nairobi, Kenya, 2007).

[50] UNEP (United Nations Environment Programme). Global Environment Outlook 5: Environment for the future we want. (United Nations Environment Programme, Nairobi, Kenya, 2012).

[51] UNEP (United Nations Environment Programme). *Global Environment Outlook – GEO-6: Healthy planet, healthy people*. (United Nations Environment Programme. Cambridge University Press, 2019).

[52] Secretariat of the Convention on Biological Diversity. Global Biodiversity Outlook 2. 81 + vii (Montréal, Canada, 2006).

[53] Secretariat of the Convention on Biological Diversity. Global Biodiversity Outlook 3. 94 (Montréal, Canada, 2010).

[54] Secretariat of the Convention on Biological Diversity. Global Biodiversity Outlook 4. 155 (Montréal, Canada, 2014).

[55] Secretariat of the Convention on Biological Diversity. Global Biodiversity Outlook 5. (Montréal, Canada, 2020).

[56] Ramsar Convention on Wetlands. Global wetland outlook: State of the world’s wetlands and their services to people. (Ramsar Convention Secretariat, Gland, Switzerland, 2018).

[57] MWO (Mediterranean Wetlands Observatory). Mediterranean Wetlands Outlook. First Mediterranean Wetlands Observatory report - Technical report. 128 (Tour du Valat, France, 2012).

[58] MWO (Mediterranean Wetlands Observatory). Mediterranean Wetlands Outlook 2: Solutions for Sustainable Mediterranean Wetlands. (Tour du Valat, France, 2018).

[59] Deinet, S. The Living Planet Index (LPI) for species listed on the CMS Appendices. Technical summary submitted to UNEP-WCMC and the CMS Secretariat. 11 (ZSL, UNEP/CMS/COP13/Doc.24/Annex 5, 2019).

[60] Latham, J., Collen, B., McRae, L. & Loh, J. The Living Planet Index for migratory species: An index of change in population abundance. 22 (ZSL/WWF, 2008).

[61] CAFF (Conservation of Arctic Flora and Fauna). *Arctic biodiversity assessment. Status and trends in Arctic biodiversity*. (Conservation of Arctic Flora and Fauna, 2013).

[62] Dasgupta, P. The economics of biodiversity: The Dasgupta review. (HM Treasury, London, 2021).

[63] UK Parliament.Early Day Motions. Number 624: Global biodiversity. Tabled 31st October, 2016 (2016-17 Session) Available at https://edm.parliament.uk/early-day-motion/49877/global-biodiversity (2016).

[64] McRae, L. et al. Living Planet Index Guidance for national and regional use Version 1.1. 11 (Cambridge, UK., 2008).

[65] Brooks, T. M. et al. Analysing biodiversity and conservation knowledge products to support regional environmental assessments. *Sci Data* **3**, 160007 (2016). 26881749 10.1038/sdata.2016.7 PMC4755129

[66] PACA (Observatoire Régional de la Biodiversité en Provence-Alpes-Côte d’Azur). Indice Région Vivante. Comment évolue la biodiversité en Provence-Alpes-Côte d’Azur?, (Observatoire Régional de la Biodiversité en Provence-Alpes-Côte d’Azur, 2018).

[67] Bayraktarov, E. et al. A threatened species index for Australian birds. *Conservation Science and Practice* **3**, 10.1111/csp2.322 (2020).

[68] Marconi, V. et al. Population declines among Canadian vertebrates: But data of different quality show diverging trends. *Ecol. Indicators* **130**, 108022 (2021).

[69] Environment and Climate Change Canada (ECCC). Canadian Environmental Sustainability Indicators: Canadian species index. (2019).

[70] Freeman, R., McRae, L., Deinet, S., Amin, R. & Collen, B. *rlpi: Tools for calculating indices using the Living Planet Index method. R Package*, https://github.com/Zoological-Society-of-London/rlpi (2017).

[71] ICMBio-CENAP/Programa-Monitora-Florestal-Global. *Analise de dados Mastoaves do protocolo florestal global do programa Monitora*, https://github.com/ICMBio-CENAP/Programa-Monitora-Florestal-Global (2021).

[72] Moreno, I., Gippet, J. M. W., Fumagalli, L. & Stephenson, P. J. Factors affecting the availability of data on East African wildlife: the monitoring needs of conservationists are not being met. *Biodivers. Conserv.* **32**, 249–273 (2022).

[73] GBIF. *GBIF: The Global Biodiversity Information Facility*, https://www.gbif.org/ (2021).

[74] IUCN. *The IUCN Red List of Threatened Species. Version 2021-3*. https://www.iucnredlist.org (2021).

[75] Hudson, L. N. et al. The database of the PREDICTS (Projecting Responses of Ecological Diversity In Changing Terrestrial Systems) project. *Ecol. Evol.* **7**, 145–188 (2017). 28070282 10.1002/ece3.2579 PMC5215197

[76] Dornelas, M. et al. BioTIME: A database of biodiversity time series for the Anthropocene. *Glob. Ecol. Biogeogr.* **27**, 760–786 (2018). 30147447 10.1111/geb.12729 PMC6099392

[77] Geldmann, J. et al. A global analysis of management capacity and ecological outcomes in terrestrial protected areas. *Conserv. Lett.* **11**, e12434 (2018).

[78] Craigie, I. D. et al. Large mammal population declines in Africa’s protected areas. *Biol. Conserv.* **143**, 2221–2228 (2010).

[79] Barnes, M. D. et al. Wildlife population trends in protected areas predicted by national socio-economic metrics and body size. *Nat. Commun.* **7**, 12747 (2016). 27582180 10.1038/ncomms12747 PMC5025815

[80] Collen, B. et al. Predicting how populations decline to extinction. *Phil. Trans. R. Soc. B* **366**, 2577–2586 (2011). 21807738 10.1098/rstb.2011.0015 PMC3138608

[81] Green, E. J. et al. Below the canopy: global trends in forest vertebrate populations and their drivers. *Proc. Biol. Sci.* **287**, 20200533 (2020). 32486986 10.1098/rspb.2020.0533 PMC7341944

[82] Hardesty-Moore, M. et al. Migration in the Anthropocene: how collective navigation, environmental system and taxonomy shape the vulnerability of migratory species. *Philos Trans. R Soc. Lond. B Biol. Sci.* **373**, 10.1098/rstb.2017.0017 (2018). 10.1098/rstb.2017.0017 PMC5882986 29581401

[83] He, F. et al. The global decline of freshwater megafauna. *Glob. Chang. Biol.* **25**, 3883–3892 (2019). 31393076 10.1111/gcb.14753

[84] Saha, A. et al. Tracking Global Population Trends: Population time-series data and a living planet index for reptiles. *J. Herpetol.* **52**, 10.1670/17-076 (2018).

[85] Costelloe, B. et al. Global biodiversity indicators reflect the modeled impacts of protected area policy change: Biodiversity indicators and protected areas. *Conserv. Lett.* **9**, 14–20 (2016).

[86] Currie, J., Marconi, V. & Kerr, J. An analysis of threats and factors that predict trends in Canadian vertebrates designated as at-risk. *Facets* **5**, 49–66 (2020).

[87] Di Fonzo, M. D., Collen, B. & Mace, G. M. A new method for identifying rapid decline dynamics in wild vertebrate populations. *Ecol. Evol.* **3**, 2378–2391 (2013). 23919177 10.1002/ece3.596 PMC3728972

[88] McRae, L. et al. A global indicator of utilized wildlife populations: Regional trends and the impact of management. *One Earth* **5**, 422–433 (2022).

[89] Capdevila, P., Noviello, N., McRae, L., Freeman, R. & Clements, C. F. Global patterns of resilience decline in vertebrate populations. *Ecol. Lett.* **25**, 240–251 (2022). 34784650 10.1111/ele.13927

[90] Spooner, F. E. B., Pearson, R. G. & Freeman, R. Rapid warming is associated with population decline among terrestrial birds and mammals globally. *Glob. Chang. Biol.* **24**, 4521–4531 (2018). 30033551 10.1111/gcb.14361

[91] Ament, J. M. et al. Compatibility between agendas for improving human development and wildlife conservation outside protected areas: Insights from 20 years of data. *People Nat.* **1**, 305–316 (2019). 10.1002/pan3.10041 PMC8641387 34901763

[92] University of Edinburgh. *Our Coding Club*, https://ourcodingclub.github.io/ (2021).

[93] Cornford, R. et al. Fast, scalable, and automated identification of articles for biodiversity and macroecological datasets. *Glob. Ecol. Biogeogr.* **30**, 339–347 (2020).

[94] Darrah, S. E. et al. Improvements to the Wetland Extent Trends (WET) index as a tool for monitoring natural and human-made wetlands. *Ecol. Indicators* **99**, 294–298 (2019).

[95] Dixon, M. J. R. et al. Tracking global change in ecosystem area: The Wetland Extent Trends index. *Biol. Conserv.* **193**, 27–35 (2016).

[96] Harmon, D. & Loh, J. The index of linguistic diversity: A new quantitative measure of trends in the status of the world’s languages. *Lang. Document. Conserv.* **4**, 97–151 (2010).

[97] Oppenheimer, P. et al. The SPOTT index: A proof-of-concept measure for tracking public disclosure in the palm oil industry. *Curr. Res. Environ. Sustain.* **3**, 10.1016/j.crsust.2021.100042 (2021).

[98] Millard, J. W., Gregory, R. D., Jones, K. E. & Freeman, R. The species awareness index as a conservation culturomics metric for public biodiversity awareness. *Conserv. Biol.* **35**, 472–482 (2021). 33749018 10.1111/cobi.13701

[99] Rodrigues, A. S., Pilgrim, J. D., Lamoreux, J. F., Hoffmann, M. & Brooks, T. M. The value of the IUCN Red List for conservation. *Trends Ecol. Evol.* **21**, 71–76 (2006). 16701477 10.1016/j.tree.2005.10.010

[100] Turnhout, E. & Purvis, A. Biodiversity and species extinction: categorisation, calculation, and communication. *Griffith Law Rev.* **29**, 669–685 (2021).

[101] Kays, R., McShea, W. J., Wikelski, M. & Zurell, D. Born‐digital biodiversity data: Millions and billions. *Diversity Distributions* **26**, 644–648 (2020).

[102] Ancrenaz, M. et al. Aerial surveys give new estimates for orangutans in Sabah, Malaysia. *PLoS Biol.* **3**, e3 (2005). 15630475 10.1371/journal.pbio.0030003 PMC534813

[103] Collen, B., Ram, M., Zamin, T. & McRae, L. The tropical biodiversity data gap: Addressing disparity in global monitoring. *Trop. Conserv. Sci.* **1**, 75–88 (2008).

[104] Moussy, C. et al. A quantitative global review of species population monitoring. *Conserv. Biol*. 10.1111/cobi.13721 (2021). 10.1111/cobi.13721 33595149

[105] Proença, V. et al. Global biodiversity monitoring: From data sources to Essential Biodiversity Variables. *Biol. Conserv.* **213**, 256–263 (2017).

[106] Hoffmann, M., Brooks, T. M., Butchart, S. H. M., Gregory, R. D. & McRae, L. in *Encyclopedia of the Anthropocene* 175-184 (2018).

[107] Stephenson, P. J. et al. Priorities for big biodiversity data. *Front. Ecol. Environ.* **15**, 124–125 (2017).

[108] WWF. Living planet Report 2020. Bending the curve of biodiversity loss: A deep dive into the Living Planet Index. Marconi, V., McRae, L., Deinet, S., Ledger, S. and Freeman, F. in (WWF, Gland, Switzerland, 2020).

[109] Amano, T. & Sutherland, W. J. Four barriers to the global understanding of biodiversity conservation: wealth, language, geographical location and security. *Proc. Biol. Sci.* **280**, 20122649 (2013). 23390102 10.1098/rspb.2012.2649 PMC3574366

[110] Montgomery, S. L. *Does science need a global language?*, (The University of Chicago Press, 2013).

[111] Amano, T., Gonzalez-Varo, J. P. & Sutherland, W. J. Languages Are Still a Major Barrier to Global Science. *PLoS Biol.* **14**, e2000933 (2016). 28033326 10.1371/journal.pbio.2000933 PMC5199034

[112] WWF. Living Planet Report 2022 – Building a nature-positive society. (WWF, Gland, Switerland, 2022).

[113] Wilkinson, M. D. et al. The FAIR Guiding Principles for scientific data management and stewardship. *Sci. Data* **3**, 160018 (2016). 26978244 10.1038/sdata.2016.18 PMC4792175

[114] Buckland, S. T., Magurran, A. E., Green, R. E. & Fewster, R. M. Monitoring change in biodiversity through composite indices. *Phil. Trans. R. Soc. B* **360**, 243–254 (2005). 15814343 10.1098/rstb.2004.1589 PMC1569463

[115] Buckland, S. T., Marsden, S. J. & Green, R. E. Estimating bird abundance: making methods work. *Bird Conserv. Int.* **18**, S91–S108 (2008).

[116] Gregory, R. D. et al. Developing indicators for European birds. *Philos. Trans. R Soc. Lond. B Biol. Sci.* **360**, 269–288 (2005). 15814345 10.1098/rstb.2004.1602 PMC1569455

[117] Buckland, S. T., Studeny, A. C., Magurran, A. E., Illian, J. B. & Newson, S. E. The geometric mean of relative abundance indices: a biodiversity measure with a difference. *Ecosphere* **2**, 10.1890/es11-00186.1 (2011).

[118] Gregory, R. D., Skorpilova, J., Vorisek, P. & Butler, S. An analysis of trends, uncertainty and species selection shows contrasting trends of widespread forest and farmland birds in Europe. *Ecol. Indicators* **103**, 676–687 (2019).

[119] Leung, B. et al. Clustered versus catastrophic global vertebrate declines. *Nature* **588**, 267–271 (2020). 33208939 10.1038/s41586-020-2920-6

[120] Santini, L. et al. Assessing the suitability of diversity metrics to detect biodiversity change. *Biol. Conserv.* **213**, 341–350 (2017).

[121] van Strien, A. J., Soldaat, L. L. & Gregory, R. D. Desirable mathematical properties of indicators for biodiversity change. *Ecol. Indicators* **14**, 202–208 (2012).

[122] Puurtinen, M., Elo, M. & Kotiaho, J. S. The Living Planet Index does not measure abundance. *Nature* **601**, E14–E15 (2022). 35082408 10.1038/s41586-021-03708-8

[123] van Strien, A. J. et al. Modest recovery of biodiversity in a western European country: The Living Planet Index for the Netherlands. *Biol. Conserv.* **200**, 44–50 (2016).

[124] Auger‐Méthé, M. et al. A guide to state–space modeling of ecological time series. *Ecol. Monogr.* **91**, 10.1002/ecm.1470 (2021).

[125] Daskalova, G. N., Myers-Smith, I. H. & Godlee, J. L. Rare and common vertebrates span a wide spectrum of population trends. *Nat. Commun.* **11**, 4394 (2020). 32879314 10.1038/s41467-020-17779-0 PMC7468135

[126] Bland, L. M., Collen, B., Orme, C. D. & Bielby, J. Predicting the conservation status of data-deficient species. *Conserv. Biol.* **29**, 250–259 (2015). 25124400 10.1111/cobi.12372

[127] Jaspers, A. Can a single index track the state of global biodiversity? *Biol. Conserv.* **246**, 108524 (2020).

[128] DEFRA. UK Biodiversity indicators 2022. (Department for Environment, Food and Rural Affairs, UK, 2022).

[129] Hansen, M. C. et al. High-Resolution Global Maps of 21st-Century Forest Cover Change. *Science* **342**, 850–853 (2013). 24233722 10.1126/science.1244693

[130] Kinnebrew, E. et al. Biases and limitations of Global Forest Change and author-generated land cover maps in detecting deforestation in the Amazon. *PLoS ONE* **17**, e0268970 (2022). 35793333 10.1371/journal.pone.0268970 PMC9258877

[131] Tropek, R. et al. Comment on “High-resolution global maps of 21st-century forest cover change”. *Science* **344**, 981–981 (2014). 24876487 10.1126/science.1248753

[132] Martin, L. J., Blossey, B. & Ellis, E. Mapping where ecologists work: biases in the global distribution of terrestrial ecological observations. *Front. Ecol. Environ.* **10**, 195–201 (2012).

[133] Higgs, E. et al. The changing role of history in restoration ecology. *Front. Ecol. Environ.* **12**, 499–506 (2014).

[134] Schulte To Buhne, H., Pettorelli, N. & Hoffmann, M. The policy consequences of defining rewilding. *Ambio* **51**, 93–102 (2022). 33983560 10.1007/s13280-021-01560-8 PMC8651963

[135] Collins, A. C., Böhm, M. & Collen, B. Choice of baseline affects historical population trends in hunted mammals of North America. *Biol. Conserv.* **242**, 10.1016/j.biocon.2020.108421 (2020).

[136] Navarro, M. & Tidball, K. G. Challenges of biodiversity education: A review of education strategies for conserving biodiversity. *Int. Electronic J. Environ. Educ.* **2**, 13–30 (2012).

[137] Carrington, D. *Humanity has wiped out 60% of animal populations since 1970, report finds*, https://www.theguardian.com/environment/2018/oct/30/humanity-wiped-out-animals-since-1970-major-report-finds (2018).

[138] O’Neill, S. & Nicholson-Cole, S. “Fear won’t do it”: Promoting positive engagement with climate change through visual and iconic representations. *Sci. Commun.* **30**, 355–379 (2009).

[139] Freeman, R. *The Living Planet Index – data analysis, clusters and biodiversity loss*, https://www.zsl.org/blogs/science/the-living-planet-index--data-analysis-clusters-and-biodiversity-loss (2020).

[140] Buschke, F. T., Hagan, J. G., Santini, L. & Coetzee, B. W. T. Random population fluctuations bias the Living Planet Index. *Nat. Ecol. Evol.* **5**, 1145–1152 (2021). 34168337 10.1038/s41559-021-01494-0

[141] Ritchie, H. *Living Planet Index*, https://ourworldindata.org/living-planet-index (2021).

[142] Hayhow, D. B. et al. State of nature 2019. (State of Nature Partnership, 2019).

[143] Hochkirch, A. et al. A strategy for the next decade to address data deficiency in neglected biodiversity. *Conserv Biol* **35**, 502–509 (2021). 32656858 10.1111/cobi.13589

[144] International Research Coordination Network. *Status of Insects project*, https://statusofinsects.github.io/ (2023).

[145] Antonelli, A. et al. State of the World’s Plants and Fungi 2020. (Royal Botanic Gardens, Kew, 2020).

[146] Henriques, S. et al. Accelerating the monitoring of global biodiversity: Revisiting the sampled approach to generating Red List Indices. *Conserv. Lett.* **13**, 10.1111/conl.12703 (2020).

[147] Brummitt, N. A. et al. Green Plants in the Red: A Baseline Global Assessment for the IUCN Sampled Red List Index for Plants. *PLoS ONE* **10**, 10.1371/journal.pone.0135152 (2015). 10.1371/journal.pone.0135152 PMC4529080 26252495

[148] Baillie, J. E. M. et al. Toward monitoring global biodiversity. *Conservation Letters* **1**, 18–26 (2008).

[149] Outhwaite, C. L., Gregory, R. D., Chandler, R. E., Collen, B. & Isaac, N. J. B. Complex long-term biodiversity change among invertebrates, bryophytes and lichens. *Nature Ecology & Evolution* **4**, 384–392 (2020). 32066888 10.1038/s41559-020-1111-z

[150] Pocock, M. J. O., Logie, M. W., Isaac, N. J. B., Outhwaite, C. L. & August, T. Rapid assessment of the suitability of multi-species citizen science datasets for occupancy trend analysis. Preprint at 10.1101/813626 (2019).

[151] van Strien, A. J., van Swaay, C. A. M., van Strien-van Liempt, W. T. F. H., Poot, M. J. M. & WallisDeVries, M. F. Over a century of data reveal more than 80% decline in butterflies in the Netherlands. *Biol. Conser.* **234**, 116–122 (2019).

[152] Mouquet, N. et al. REVIEW: Predictive ecology in a changing world. *J. Appl. Ecol.* **52**, 1293–1310 (2015).

[153] Visconti, P. et al. Projecting Global Biodiversity Indicators under Future Development Scenarios. *Conserv. Lett.* **9**, 5–13 (2016).

[154] Cardoso, P., Stoev, P., Georgiev, T., Senderov, V. & Penev, L. Species Conservation Profiles compliant with the IUCN Red List of Threatened Species. *Biodivers Data J*, e10356, 10.3897/BDJ.4.e10356 (2016). 10.3897/BDJ.4.e10356 PMC5018116 27660524

[155] Grames, E. et al. Trends in global insect abundance and biodiversity: A community-driven systematic map protocol. *Open Science Framework (osf.io/uxk4a)*10.17605/OSF.IO/Q63UY (2019).

[156] Amano, T. et al. Tapping into non-English-language science for the conservation of global biodiversity. *PLoS Biol.* **19**, e3001296 (2021). 34618803 10.1371/journal.pbio.3001296 PMC8496809

[157] McRae, L., Deinet, S. & Freeman, R. LPI_LPR2016data_public.csv. *Figshare. Dataset*, 10.6084/m9.figshare.4300022.v1 (2016).

[158] UNEP-WCMC & IUCN. *Protected Planet*, https://www.protectedplanet.net/en (2021).

[159] Jellesmark, S. et al. A counterfactual approach to measure the impact of wet grassland conservation on U.K. breeding bird populations. *Conserv. Biol.* **35**, 1575–1585 (2021). 33415751 10.1111/cobi.13692

[160] Salafsky, N. et al. A standard lexicon for biodiversity conservation: unified classifications of threats and actions. *Conserv. Biol.* **22**, 897–911 (2008). 18544093 10.1111/j.1523-1739.2008.00937.x

[161] Bowles-Newark, N. J., Chenery, A., Misrachi, M. & Despot-Belmonte, K. Barriers to the use of global indicators and datasets to support NBSAP implementation and national reporting processes. (UNEP-WCMC, Cambridge, UK, 2015).

[162] Butchart, S. H. et al. Improvements to the Red List Index. *PLoS ONE* **2**, e140 (2007). 17206275 10.1371/journal.pone.0000140 PMC1764037

[163] Hill, S. L. L. et al. Worldwide impacts of past and projected future land-use change on local species richness and the Biodiversity Intactness Index. Preprint at 10.1101/311787 (2018).

[164] Soto-Navarro, C. A. et al. Building a Multidimensional Biodiversity Index – A scorecard for biodiversity health. Project report. (UN Environment Programme World Conservation Monitoring Centre (UNEP-WCMC), Cambridge, UK and Luc Hoffmann Institute (LHI), Gland, Switzerland., 2020).

[165] Soto-Navarro, C. A. et al. Towards a multidimensional biodiversity index for national application. *Nat. Sustainability* **4**, 933–942 (2021).

[166] Brotons, L. et al. Estat de la Natura a Catalunya 2020. (Departament de Territori i Sostenibilitat. Generalitat de Catalunya, Barcelona, 2020).

[167] Maas, S. & Giroud, I. Indice Région Vivante (IRV): indicateurs oiseaux de Franche-Comté. 11p (LPO Franche-Comté, DREAL Bourgogne Franche-Comté et Conseil Régional Bourgogne Franche-Comté, 2016).

[168] Pomeroy, D., Tushabe, H. & Loh, J. The State of Uganda’s Biodiversity 2017. (National Biodiversity Data Bank. Makerere University, Kampala, 2017).

[169] Pomeroy, D. & Tushabe, H. The State of Uganda’s Biodiversity 2006. (Makerere Institute of Environment and Natural Resources/National Biodiversity Data Bank, 2006).

[170] NEMA (National Environment Management Authority). State of Environment Report for Uganda. 332 (NEMA, Kampala, Uganda, 2006/7).

[171] Pomeroy, D. & Tushabe, H. The state of Uganda’s biodiversity report: Sixth biennial report. (National Biodiversity Data Bank (NBDB), Makerere University Institute of Environment and Natural Resources (MUIENR), 2008).

[172] WWF-Norge. Naturindeks for Norge 2005. Utfor bakke med norsk natur. (WW-Norge, Oslo, Norway, 2005).

[173] Muller, H. et al. The Canadian Species Index. (ZSL/Environment Canada, 2016).

[174] CBS, PBL, RIVM & WUR. *Trend fauna - all species monitored - Living Planet Index Netherlands, 1990-2018 (indicator 1569, version 05, 30 March 2020)*, https://www.clo.nl/en/indicators/en1569-living-planet-index-for-the-netherlands (2021).

[175] WWF China. Living planet report China 2015: Development, species and ecological civilization. (WWF China in partnership with China Council for International Cooperation on Environment and Development (CCICED), Institute of Geographic Sciences and Natural Resources Research (IGSNRR) and Institute of Zoology of Chinese Academy of Sciences (CAS), and the Global Footprint Network, 2015).

[176] The Government of China. Sixth national report to the Conventional on Biological Diversity. (Secretariat of the Convention on Biological Diversity (SCBD), The Clearing-House Mechanism of the Convention on Biological Diversity (CHM), 2019).

[177] Semmelmayer, K. & Hackländer, K. Monitoring vertebrate abundance in Austria: Developments over 30 years. *Die Bodenkultur: J. Land Manag., Food, Environ.* **71**, 19–30 (2020).

[178] McRae, L., Böhm, M., Deinet, S., Gill, M. & Collen, B. The Arctic Species Trend Index: using vertebrate population trends to monitor the health of a rapidly changing ecosystem. *Biodiversity* **13**, 144–156 (2012).

[179] McRae, L. et al. Arctic Species Trend Index 2010. Tracking Trends in Arctic Wildlife. (CAFF International Secretariat, 2010).

[180] McRae, L., Deinet, S., Gill, M. & Collen, B. The Arctic Species Trend Index: Tracking trends in Arctic marine populations. (Conservation of Arctic Flora and Fauna (CAFF), Iceland, 2012).

[181] Deinet, S. et al. Arctic Species Trend Index: Migratory Birds Index. (Conservation of Arctic Flora and Fauna (CAFF), Akureyri, Iceland, 2015).

[182] Galewski, T., Segura, L., Biquet, J., Saccon, E. & Boutry, N. Living Mediterranean Report: Monitoring species trends to secure one of the major biodiversity hotspots. (Tour du Valat (TdV), France, 2021).

[183] EEA (European Environment Agency). Marine messages II: Navigating the course towards clean, healthy and productive seas through implementation of an ecosystem‑based approach. (European Environment Agency, Luxembourg, 2019).

[184] Deinet, S. et al. The Living Planet Index for Global Estuarine Systems: Technical report. (WWF/ZSL, 2010).

[185] WWF. Living planet report 2016. Risk and resilience in a new era. Report No. 978-2-940529-40-7, (WWF, Gland, Switzerland, 2016).

[186] Deinet, S. et al. The Living Planet Index (LPI) for migratory freshwater fish - Technical Report. (World Fish Migration Foundation, The Netherlands, 2020).

[187] Tierney, M. et al. Use it or lose it: Measuring trends in wild species subject to substantial use. *Oryx* **48**, 420–429 (2014).

[188] Pacoureau, N. et al. Half a century of global decline in oceanic sharks and rays. *Nature* **589**, 567–571 (2021). 33505035 10.1038/s41586-020-03173-9

[189] Loh, J. & Harmon, D. Biocultural Diversity: threatened species, endangered languages. (WWF Netherlands, Zeist, The Netherlands, 2014).

[190] UNEP-WCMC (UN Environment Programme World Conservation Monitoring Centre). *The Biodiversity Indicators Partnership (BIP)* https://www.bipindicators.net/ (2021).

[191] UNEP (United Nations Environment Programme). Decision X/2: The strategic plan for biodiversity 2011–2020 and the Aichi Biodiversity Targets. Adopted at the 10th Conference Of The Parties (COP) to the Convention On Biological Diversity (CBD). (UNEP, Montreal, Canada, 2010).

[192] WWF/ZSL. *The Living Planet Index Database (LPD)*, www.livingplanetindex.org (2022).

